# Role of Contrast-Enhanced Ultrasound (CEUS) in the Diagnosis of Cervical Lymph Node Metastasis in Nasopharyngeal Carcinoma (NPC) Patients

**DOI:** 10.3389/fonc.2020.00972

**Published:** 2020-07-17

**Authors:** Wenwu Ling, Ji Nie, Dingyue Zhang, Qianru Yang, Hongyu Jin, Xuejin Ou, Xuelei Ma, Yan Luo

**Affiliations:** ^1^Department of Ultrasound, West China Hospital, Sichuan University, Chengdu, China; ^2^Department of Biotherapy, State Key Laboratory of Biotherapy and Cancer Center, West China Hospital, Sichuan University and Collaborative Innovation Center of Biotherapy, Chengdu, China; ^3^West China School of Medicine, Sichuan University, Chengdu, China; ^4^Department of Liver Surgery, West China Hospital, Sichuan University, Chengdu, China; ^5^Department of Oncology, West China Hospital, Sichuan University, Chengdu, China

**Keywords:** contrast-enhanced ultrasonography, CEUS, nasopharyngeal carcinoma, metastatic, cervical lymph nodes

## Abstract

**Objective:** The aim of the study was to evaluate the diagnostic value of contrast-enhanced ultrasound (CEUS) in distinguishing between benign and malignant cervical lymph nodes in patients with nasopharyngeal carcinoma (NPC).

**Material and Methods:** A total of 144 NPC patients with enlarged superficial cervical lymph nodes underwent CEUS examination. The comparison of CEUS image characteristics between malignant and benign cervical lymph nodes was performed in this study as well. We analyzed parameters of the time–intensity curve (TIC), which includes time to peak (TP), area under the gamma curve (AUC), and peak intensity (PI). Furthermore, receiver operating characteristic (ROC) curve analysis was also investigated to evaluate the diagnostic value of CEUS.

**Result:** We conducted 144 lymph node examinations in total, where 64 cases were biopsy-proven benign nodules and 80 cases were biopsy-proven metastatic nodules. The vast majority of the benign nodes displayed centrifugal perfusion (96.88%, 62/64) and homogeneous enhancement (93.75%, 60/64), while most of the malignant nodes showed centripetal perfusion (92.50%, 74/80) and inhomogeneous 80.00% (64/80). In addition, quantitative analysis showed that CEUS parameters including PI, TP, and AUC in benign lymph nodes (12.51 ± 2.15, 23.79 ± 11.80, and 1110.33 ± 286.17, respectively) were significantly higher than that in the malignant nodes (10.51 ± 2.98, 16.52 ± 6.95, and 784.09 ± 340.24, respectively). The assistance of the three aforementioned parameters and CEUS image characteristics would result in an acceptable diagnostic value.

**Conclusion:** Our results suggest that imaging perfusion patterns as well as quantitative parameters obtained from CEUS provide valuable information for the evaluation of cervical lymph nodes in NPC patients.

## Introduction

Nasopharyngeal carcinoma (NPC) is a highly prevalent head and neck malignancy in southeast Asia, with an incidence rate of 20 to 50 cases per 100,000 males (1). The incidence of cervical lymph node involvement in pathologically diagnosed NPC cases is up to 85%, which is much higher than other head and neck cancers (2). Therefore, evaluation of lymph node metastasis is extremely essential for the N staging and treatment of NPC, as well as for the prognosis of NPC (3, 4). Fine needle aspiration biopsy is the gold standard for identifying cervical lymph node metastasis in NPC patients. However, due to the small amount of tissue obtained by biopsy, it is not always possible to accurately obtain the diseased tissue. With the help of imaging examination, the nature of lymph nodes can be preliminarily determined, which improves the accuracy of fine needle aspiration biopsy. Therefore, evaluation of lymph node metastasis is extremely essential for the N staging and treatment of NPC, as well as for the prognostication of NPC.

Ultrasound is one of the common tools for the diagnosis of cervical lymph nodes in NPC patients because of its cost-effectiveness and radiation-free nature (5). With gray-scale ultrasonography and Doppler imaging, malignant nodes can be differentiated from benign nodes based on various parameters such as shape, border, longitudinal-to-transverse diameter ratio, strength and distribution of echogenicity, vascularization pattern, etc. (4, 6–9). However, the diagnostic accuracy of ultrasound for lymph nodes is mediocre, and thus, a diagnostic tool with a good diagnostic value is highly demanded.

Contrast-enhanced ultrasound (CEUS) makes it possible to evaluate tissue perfusion and micro-vascularization in real time (10, 11). The use of contrast can significantly improve the diagnostic accuracy of the usual ultrasound examination. Several studies have shown that CEUS might be one potential modality for the assessment of lymph nodes (12, 13); however, the accuracy of detection of metastatic lymph nodes in NPC patients is not well-defined. Therefore, in this study, we focused on the use of CEUS to correctly identify benign and malignant lymph nodes in NPC patients.

## Methods

### Patients and Study Design

Written informed consent was signed by all patients participating in the study, and this study was approved by the Institution's Ethics Committee. From November 2014 to November 2017, 144 NPC patients who had enlarged superficial cervical lymph nodes in West China Hospital, Sichuan University were recruited into this study. Patients were included if they had undergone a biopsy for a nasopharyngeal mass and had a single lymph node or multiple lymph nodes larger than 0.5 cm. Patients with lymphoma or who were younger than 18 years of age were excluded from this study. The results were compared with the histological examination of the nodes.

### CEUS Examination

CEUS was performed using an ultrasound system (iU22; Philips Healthcare, Bothell, WA) with an L9-3 linear array transducer, a frequency of approximately 3–9 MHz, and a mechanical index of 0.06. A bolus of 2.4 ml of contrast agent (SonoVue, Milan, Italy) was injected intravenously, followed by a wash with 5 ml of saline. The wash-in and wash-out process within the lesion was dynamically observed, and the DICOM dynamic data were stored at 1 min 30 s. The arterial phase started approximately 10 to 20 s after contrast agent injection, and lasted until around 30 to 45 s, during which the degree of enhancement increased progressively. The delayed phase (or venous phase) usually after 30 to 45 s is followed by the first arrival of contrast in the lymph node, during which the adjacent jugular vein is enhanced. For patients with multiple cervical lymphadenopathy, a lymph node was randomly selected and marked before the examination, to ensure that the biopsy results corresponded to the CEUS examination results of the same lymph node.

The parametric analysis of lymph node was performed with the software QLAB (Phillips, Amsterdam, Netherlands). After selection of a region of interest (ROI), data collected from regional lymph nodes were processed automatically by the software to generate the time–intensity curve (TIC) and a series of parameters. The main parameters and data included (1) peak intensity (PI), defined as the difference between the maximum signal intensity (SI) and baseline SI in the selected ROI; (2) time to peak (TP), defined as the time it takes to reach the maximum SI from the beginning of lymph node enhancement; and (3) the area under the curve (AUC), defined as the area under the TIC.

According to the perfusion directions of contrast agents, the CEUS image patterns can be divided into centrifugal perfusion (contrast agents fill from the center to the surrounding parts) and centripetal perfusion (contrast agents fill from peripheral areas into the central regions). On the basis of the homogeneity of contrast agents in the nodules, the CEUS image patterns can be divided into homogeneous enhancement (the contrast agent is evenly distributed in the nodules) and inhomogeneous enhancement (the contrast agent is unevenly distributed in the nodules). According to the enhancement degrees of nodules compared with adjacent tissues at peak enhancement, three CEUS image patterns were defined, which are hyperenhancement (the enhancement degree is higher in nodule), hypoenhancement (the enhancement degree is lower in nodule), and no enhancement (the enhancement degree of nodule is similar to that of adjacent tissues).

The results of CEUS were analyzed by two radiologists with over 5 years of experience without the information of the pathological results. Image evaluation was performed by each radiologist separately. After analyzing the ultrasound images independently, the two radiologists reviewed the images and reached a consensus to ensure intra-investigator agreement. Analysis was repeated more than three times by the same investigator.

### Histopathological Diagnosis

Histopathological diagnosis was obtained by biopsy or surgical removal of lymph nodes after the CEUS examination. The CEUS results were compared to the histological results.

### Statistical Analysis

We analyzed the results with standard descriptive statistics, means of the numerical results were compared by *t*-test (Mann–Whitney *U*-test for non-normally distributed data), and Chi-square was adopted for the comparison of categorical parameters. The receiver operating characteristics (ROC) analysis was adopted to evaluate the accuracy of CEUS in distinguishing benign lymph nodes from malignant lymph nodes by calculating the area under the curve (we called it AUROC to avoid the confusion with the CEUS software measurement parameter AUC). Sensitivity, specificity, and overall ability were compared. Difference was considered to be significant when *P* was below 0.05. The analysis was performed with SPSS 22.0 software (IBM Corp, Armonk, NY).

## Result

### Surgery and Histology

A total of 144 NPC patients were included in this study, including 74 males and 70 females, with an average age of 51.8 ± 14.0 years (range, 21–80 years). All of them conducted both CEUS examinations and pathological diagnosis. Their histories of surgery or histological examination results in the past were also analyzed. In these cervical lymph nodes, 80 patients were confirmed as metastatic lymph nodes, while 64 patients were confirmed as benign lymph nodes.

### Characteristics of CEUS Image

**Figure 1:**
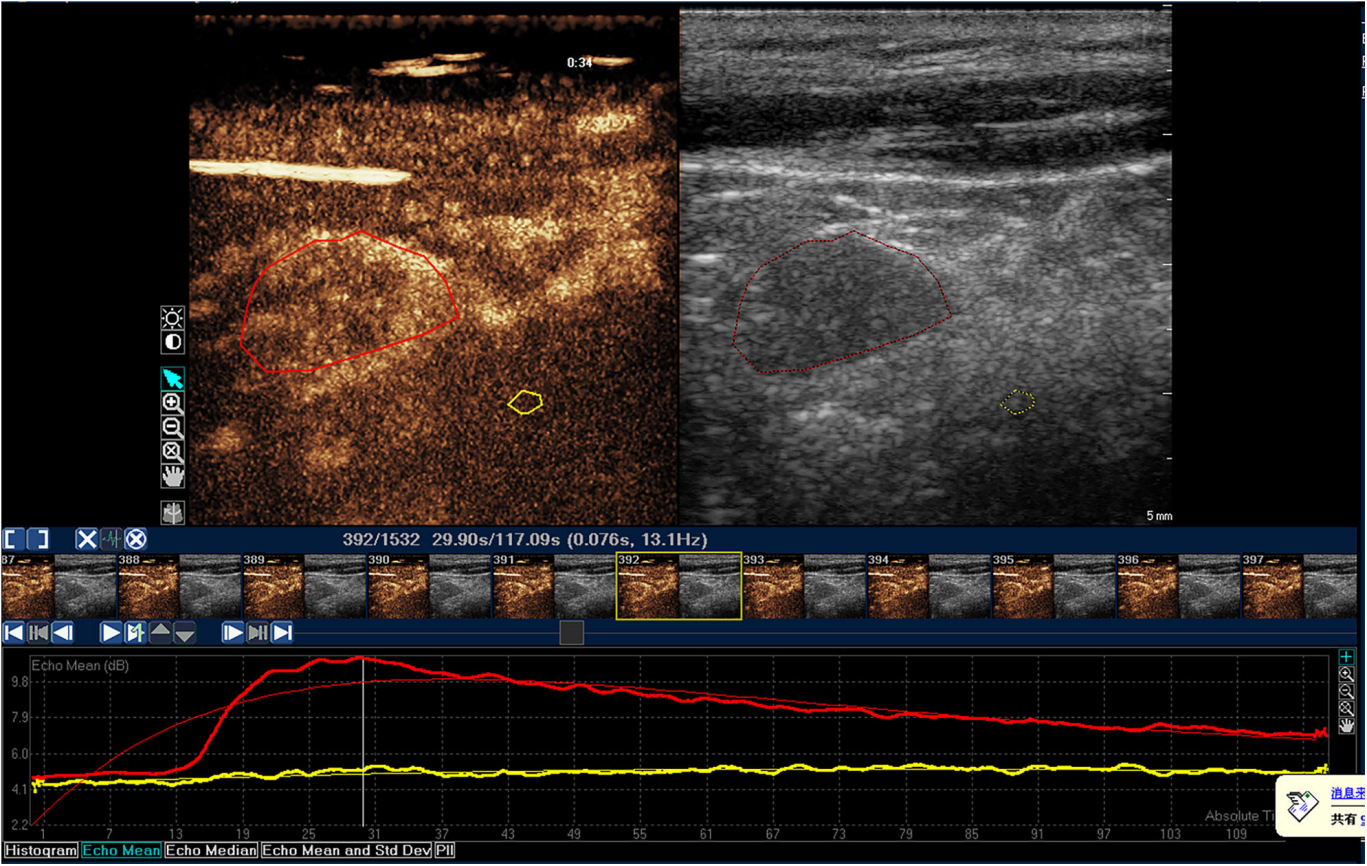
Time-intensity curve of a typical benign lymph node in NPC patient. The red ROI is benign lymph node, and the red line is corresponding TIC curve. The yellow ROI is the tissue around the lesion, and the yellow line is the corresponding TIC curve. The benign lymph node was characterized by homogeneous enhancement.

**Figure 2:**
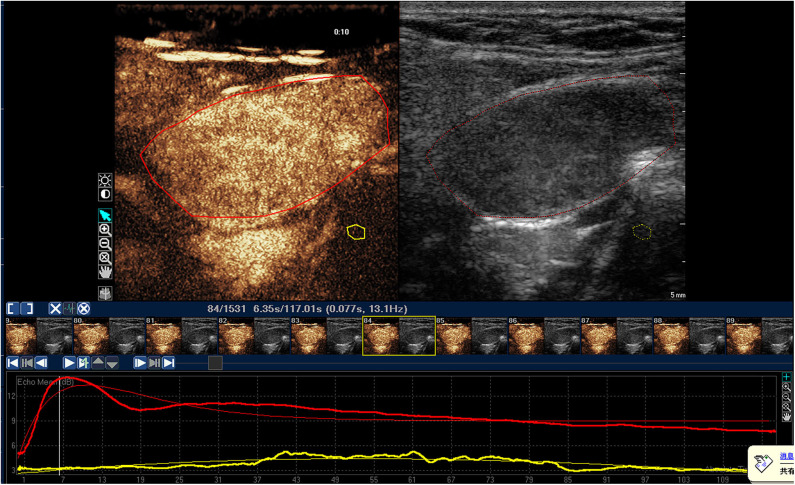
Time-intensity curve of a typical malignant lymph node in NPC patient. The red ROI is malignant lymph node, and the red line is corresponding TIC curve. The yellow ROI is the tissue around the lesion, and the yellow line is the corresponding TIC curve. The malignant lymph node was presented as inhomogeneous enhancement, and PI, TP, and AUC of malignant lymph node were significantly higher than those in benign lymph node.

The characteristics of lymph nodes obtained from CEUS (including internal lesion' s homogeneity and enhancement orders) are shown in Figures 1, 2 and listed in Table 1. Most of the metastatic lymph nodes (92.50%, 74/80) exhibited centripetal perfusion, and only 7.50% (6/80) of them showed centrifugal perfusion. Conversely, centrifugal perfusion was observed in most of the benign lymph nodes (96.88%, 62/64), and only 3.12% (2/64) of them presented as centripetal perfusion. Inhomogeneous enhancement was found in 80.00% (64/80) of metastatic lymph nodes, while most benign lymph nodes (93.75%, 60/64) showed homogeneous enhancement patterns. For the lesions' degree of enhancement, both the metastatic (97.5%, 78/80) and benign (81.25%, 52/64) nodes exhibited predominant hyperenhancement. Perfusion defects were observed only in the metastatic nodes (37.50%, 30/80). There were ring-enhancing margins in two of the metastatic lymph nodes (2.50%). The differences in the above characteristics were statistically significant (*P* all <0.001), except for the ring-enhancing margin (*P* = 0.503).

**Table 1:** Comparison of contrast-enhanced ultrasound (CEUS) image characteristics between benign and metastatic lymph nodes in NPC patients.

**Characteristics of CEUS image**	**Metastatic lymph nodes of NPC(*n* = 80)**	**Benign lymph nodes(*n* = 64)**	***P*-value**
Lesion's enhancement order			0.000
Centrifugal perfusion	6 (7.50%)	62 (96.88%)	
Centripetal perfusion	74 (92.50%)	2 (3.12%)	
Internal lesion's homogeneity			0.000
Homogeneous enhancement	16 (20.00%)	60 (93.75%)	
Inhomogeneous enhancement	64 (80.00%)	4 (6.25%)	
Enhancement degree			0.001
Hyper-enhancement	78 (97.50%)	52 (81.25%)	
Hypo-enhancement	2 (2.50%)	12 (18.75%)	
No enhancement	0	0	
Presence of perfusion defects			0.000
Yes	30 (37.50%)	0	
No	50 (62.50%)	64 (100%)	
Ring high enhancement of surrounding area			0.503
Yes	2 (2.50%)	0	
No	78 (97.50%)	64 (100%)	

### Parameters of CEUS

Comparisons of all parameters in the gamma variate are listed in Table 2. PI of benign lymph nodes (12.51 ± 2.15) was considerably higher than that of malignant nodes (10.51 ± 2.98) (*P* < 0.05). Similarly, TP was higher in benign nodes as well, while it was 23.79 ± 11.80 for benign lymph nodes and 16.52 ± 6.95 for malignant nodes (*P* < 0.05). AUC of benign lymph nodes (1110.33 ± 286.17) was also higher than that of malignant lymph nodes (784.09 ± 340.24) (*P* < 0.05).

**Table 2:** Comparison of the parameters in the contrast-enhanced ultrasound (CEUS) between benign and metastatic lymph nodes in NPC patients.

**Group**	**No. of cases**	**PI, dB**	**TP, s**	**AUC, dB × s**
Metastatic lympho nodes of NPC	80	10.51 ± 2.98	16.52 ± 6.95	784.09 ± 340.24
Benign lymph nodes	64	12.51 ± 2.15	23.79 ± 11.80	1110.33 ± 286.17
*t*-value		−4.668	−4.603	−6.129
*P*-value		0.000	0.000	0.000

### ROC Analysis

**Figure 3:**
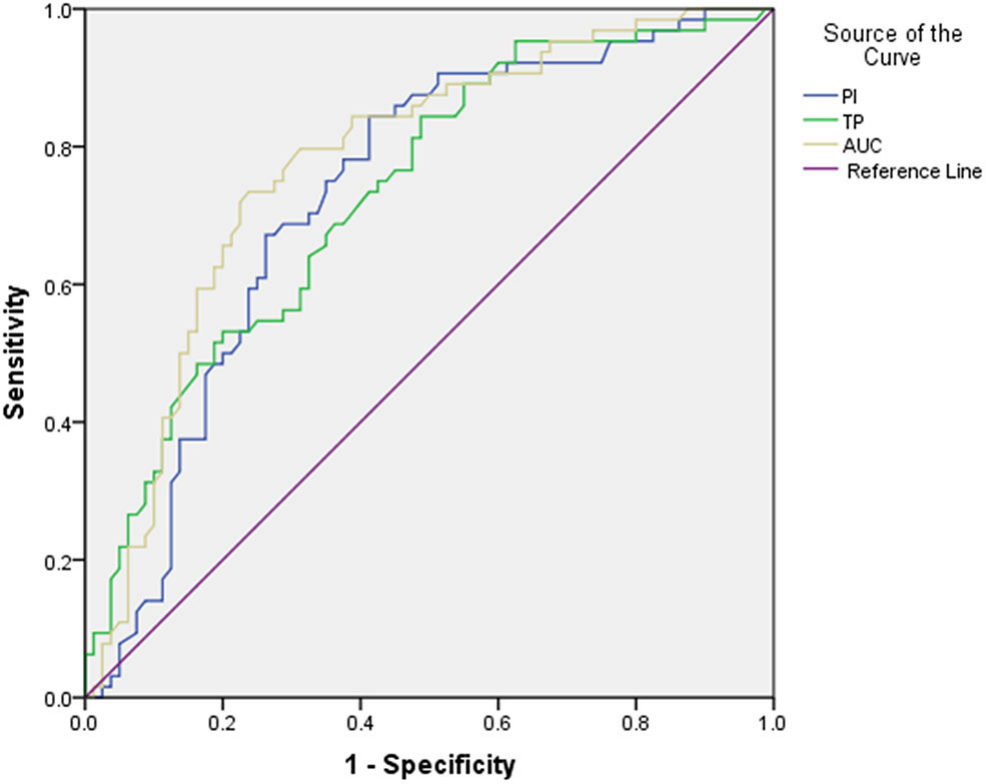
The receiver operating characteristic (ROC) curve for CEUS parameters.

To determine the capability of CEUS in distinguishing benign and malignant lymph nodes in NPC patients, ROC analysis was performed (Table 3 and Figure 3). According to our results, parameters obtained from CEUS include PI (with a cutoff value of 10.35 and an AUROC of 0.731), TP (with a cutoff value of 15.98 and an AUROC of 0.732), and AUC (with a cutoff value of 966.18 and an AUROC of 0.776). All parameters showed good diagnostic effects on NPC cervical lymph nodes.

**Table 3:** ROC analysis: benign vs. malignant nodes.

**Index**	**AUROC value**	***P*-value**	**CI 95%**	**Cut-off value**	**Sensitivity (%)**	**Specificity (%)**
PI	0.731	<0.0001	0.648–0.814	10.35	58.75	84.37
TP	0.732	<0.0001	0.65–0.813	15.98	51.25	84.37
AUC	0.776	<0.0001	0.699–0.853	966.18	76.25	73.44
PI+TP+AUC	0.795	<0.0001	0.721–0.869	\	73.75	76.56
PI+TP+AUC+Perfusion pattern	0.987	<0.0001	0.974–0.999	\	92.5	96.87
PI+TP+AUC+Enhancement pattern	0.951	<0.0001	0.918–0.984	\	87.5	90.62
PI+TP+AUC+Perfusion pattern+Enhancement pattern	0.995	<0.0001	0.988–1	\	95	96.87

**Figure 4:**
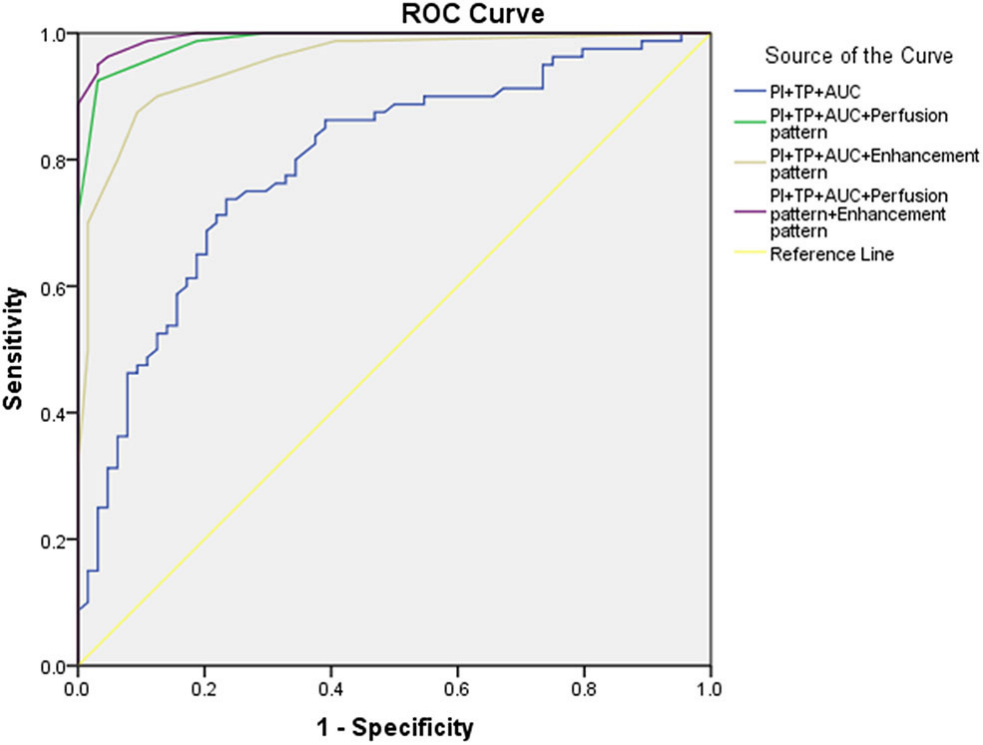
The receiver operating characteristic (ROC) curve for combination diagnosis.

Data were combined to see if a better result in the diagnosis of cervical lymph nodes can be achieved (Table 3 and Figure 4). The combination of CEUS parameters (PI, TP, and AUC) got a higher diagnostic value: sensitivity, specificity, and AUROC were 73.75%, 75.56%, and 0.795, respectively. Besides, when combining CEUS parameters with CEUS image characteristics, a better sensitivity, specificity, and AUROC can be achieved, which were 95.00%, 96.87%, and 0.995, respectively.

## Discussion

Diagnosis of the nature of lymph nodes in the head and neck has been a challenge for clinicians. Imaging modalities widely used for diagnosis include CT, MRI, and FDG-PET (14, 15). However, the diagnostic capacity of these imaging methods is limited for lymph nodes, especially when the nodes are <10 mm in diameter, or when there is no necrosis or calcification in the nodes (16). On the other hand, the resolution of lymph nodes on ultrasound is better than that of CT and MRI. Ultrasound is also cheaper (17).

With the microbubble's nature, ultrasonic contrast agent is capable to pass through the vascular system and can detect avascular areas of necrosis as well. This study demonstrated that CEUS is better capable of describing the microvascular pattern and is potentially quite clinically valuable in the description of tumor metastasis (18). Diagnosing focal liver lesions is one of the most common applications of CEUS, and many clinical studies pointed out that CEUS outperformed CT and almost the same performance as MRI in diagnosing hepatic masses (19–21). However, studies related to the use of CEUS in superficial lymph nodes are still insufficient. This study concentrated on the utility of CEUS in the differentiation of benign from malignant cervical lymph nodes. In this study, we aimed at finding characteristic imaging patterns and parameters in CEUS to distinguish between benign and metastatic cervical lymph nodes of NPC patients.

The intense homogeneous enhancement of reactive benign lymph nodes on CEUS was reported in previous studies, due to the abundant blood supply provided by the dense and uniform capillary circulation around the nodes (22). Some studies indicated that the degree of vascularization of metastatic lymph nodes was generally lower than that of reactive benign lymph nodes. As a result, malignant lymph nodes were often found to be heterogeneously hypoenhancing because of areas of perfusion defects (23, 24). In this study, similar results of this enhancement pattern were observed. The majority of benign lymph nodes (93.75%) were found to be homogeneously enhancing, either moderately or intensely, and only four cases showed inhomogeneous enhancement. On the other hand, most of malignant nodes (80%) in our study were inhomogeneous on CEUS.

According to our results, there was a significant difference in enhancement orders between benign and malignant lymph nodes: most of the metastatic nodes in this study showed a centripetal enhancement order, which can be found in only two benign lymph nodes. It appears that the lesion's enhancement pattern provides useful information for judging the nature of lymph nodes. When the CEUS image of the lymph node shows a centripetal enhancement pattern, this lymph node has high possibility to be malignant.

The absence of the echogenic hilum was usually considered to be one of the characteristics of malignancy. However, hyperechoic hilum is not often observed in cervical lymph nodes, thus making the differential diagnosis of cervical nodes challenging (25). On the other hand, the presence of a high-enhancement ring may provide important information. In our study, the high-enhancement ring around the lesions was found in some metastatic nodes, while none of the benign lesions had a high-enhancement ring. This was consistent with a previous point of view that the enhancement degree of the lesion in CEUS depended on the richness of blood supply, and the high-enhancement ring was considered to be the edge area of tumor that closely adhered to the pseudo-capsule fibrous tissue with high microvascular density (24).

Previous studies have not been consistent about the CEUS parameters of benign and malignant nodes. Some studies showed that TP and AUC of malignant lymph nodes were lower than those of benign lymph nodes, but the values of PI in these studies were still contradictory (26–28). Other studies believed that there was no significant difference in CEUS parameters (PI, TP, and AUC) between benign and malignant lymph nodes, but maximum and minimum signal intensity (simax–simin) were considered to be different (24). In our study, parameters of malignant nodes including PI, TP, and AUC were all lower than those of benign lymph nodes in NPC patients. Blood flow of some metastatic lesions might be reduced due to vascular compression caused by tumor tissue; this provided a possible explanation for various PIs of malignant lymph nodes in different researches. To obtain more reliable results, additional studies with more cases should be conducted.

According to the results of ROC analysis, CEUS parameters showed good diagnostic value in differentiating benign cervical lymph nodes from malignant ones in NPC patients. However, based on these parameters, satisfactory diagnostic results are still not obtained. When combining CEUS parameters with image features, a better diagnostic efficacy was obtained.

We recognize that there are limitations in our study. Firstly, the number of cases included in this study was limited. Secondly, some intrinsic limitations of CEUS were not considered completely. For example, imaging results obtained from this examination depend on the subjective evaluation of the clinician, and the panoramic view of both metastatic lesions and primary tumor cannot be displayed by the ultrasonic examination.

## Conclusion

In conclusion, the results of this study demonstrate the good diagnostic value of CEUS parameters in differentiating cervical benign nodes from malignant ones in NPC patients. It is recommended to perform an analysis of quantitative parameters obtained from CEUS, which provides important information for the diagnosis of cervical lymph nodes. Further investigation is required to determine the specific clinical role of CEUS in characterization of lymph nodes in NPC patients.

## Data Availability Statement

All datasets generated for this study are included in the article/supplementary material.

## Ethics Statement

The studies involving human participants were reviewed and approved by Biomedical Ethics Sub-Committee, West China Hospital, Sichuan University. The patients/participants provided their written informed consent to participate in this study. Written informed consent was obtained from the individual(s) for the publication of any potentially identifiable images or data included in this article.

## Author Contributions

The conception and design of the study: WL, JN, XM, and YL. Acquisition of data: JN and WL. Analysis and interpretation of data: QY, HJ, DZ, and XO. Drafting the article: WL and JN. The article: XM. Final approval of the version to be submitted: XM and YL.

## Conflict of Interest

The authors declare that the research was conducted in the absence of any commercial or financial relationships that could be construed as a potential conflict of interest.
